# Application of Cloud Model in Qualitative Forecasting for Stock Market Trends

**DOI:** 10.3390/e22090991

**Published:** 2020-09-06

**Authors:** Oday A. Hassen, Saad M. Darwish, Nur A. Abu, Zaheera Z. Abidin

**Affiliations:** 1 Ministry of Education, Wasit Education Directorate, Kut 52001, Iraq; odayali@uowasit.edu.iq; 2 Department of Information Technology, Institute of Graduate Studies and Research, Alexandria University, 163 Horreya Avenue, El–Shatby, Alexandria 21526, Egypt; 3 Faculty of Information and Communication Technology, University Teknikal Malaysia Melaka, Melaka 76100, Malaysia; nura@utem.edu.my (N.A.A.); zaheera@utem.edu.my (Z.Z.A.)

**Keywords:** cloud model, fuzzy time series, stock trend, Heikin–Ashi candlestick

## Abstract

Forecasting stock prices plays an important role in setting a trading strategy or determining the appropriate timing for buying or selling a stock. The use of technical analysis for financial forecasting has been successfully employed by many researchers. The existing qualitative based methods developed based on fuzzy reasoning techniques cannot describe the data comprehensively, which has greatly limited the objectivity of fuzzy time series in uncertain data forecasting. Extended fuzzy sets (e.g., fuzzy probabilistic set) study the fuzziness of the membership grade to a concept. The cloud model, based on probability measure space, automatically produces random membership grades of a concept through a cloud generator. In this paper, a cloud model-based approach was proposed to confirm accurate stock based on Japanese candlestick. By incorporating probability statistics and fuzzy set theories, the cloud model can aid the required transformation between the qualitative concepts and quantitative data. The degree of certainty associated with candlestick patterns can be calculated through repeated assessments by employing the normal cloud model. The hybrid weighting method comprising the fuzzy time series, and Heikin–Ashi candlestick was employed for determining the weights of the indicators in the multi-criteria decision-making process. Fuzzy membership functions are constructed by the cloud model to deal effectively with uncertainty and vagueness of the stock historical data with the aim to predict the next open, high, low, and close prices for the stock. The experimental results prove the feasibility and high forecasting accuracy of the proposed model.

## 1. Introduction

Forecasting stock prices is an attractive pursuit for investors and researchers who want to beat the stock market. The benefits of having a good estimation of the stock market behavior are well-known, minimizing the risk of investment and maximizing profits. Recently, the stock market has become an easily accessible investment tool, not only for strategic investors, but also for ordinary people. Over the years, investors and researchers have been interested in developing and testing models of stock price behavior. However, analyzing stock market movements and price behaviors is extremely challenging because of the market’s dynamic, nonlinear, non–stationary, nonparametric, noisy, and chaotic nature [1]. Stock markets are affected by many highly interrelated uncertain factors that include economic, political, psychological, and company-specific variables. These uncertain factors are undesirable for the stock investor and make stock price prediction very difficult, but at the same time, they are also unavoidable whenever stock trading is preferred as an investment tool [1,2]. To invest in stocks and achieve high profits with low risks, investors have used technical and fundamental analysis as two major approaches in decision-making in financial markets [2].

Fundamental analysis studies all of the factors that have an impact on the stock price of the company in the future such as financial statements, management processes, industry, etc. It analyzes the intrinsic value of the firm to identify whether the stock is underpriced or overpriced. On the other hand, technical analysis uses past charts, patterns, and trends to forecast the price movements of the entity in the coming time [2,3]. The main weakness of fundamental analysis is that it is time-consuming as people cannot quickly locate and absorb the information needed to make thoughtful stock picks. People’s judgments are subjective, as is their definition of fair value. The second drawback of a fundamental analysis is in relation to the efficient market hypothesis. Since all information about stocks is public knowledge—barring illegal insider information—stock prices reflect that knowledge.

A major advantage of technical analysis is its simple logic and application. It is seen in the fact that it ignores all economic, market, technological, and any other factors that may have an impact on the company and the industry and only focuses on the data on prices and the volume traded to estimate future prices. The second advantage of technical analysis is that it excludes the subjective aspects of certain companies such as the analyst’s personal expectations [4]. However, technical analysis may get an investor trapped: when price movements are artificially created to lure an investor into the stock and once enough investors are entered, they start selling, and you may be trapped. Furthermore, it is too reliant on mathematics and patterns in the chart of the stock and ignores the underlying reasons or causes of price movements. As a result, the stock movements are too wild to handle or predict through technical analysis.

There exist two types of forecasting techniques to be implemented [5,6]: (a) qualitative forecasting models; and (b) quantitative forecasting models. The qualitative forecasting models are generally subjective in nature and are mostly based on the opinions and judgments of experts. Such types of methods are generally used when there is little or no past data available that can be used to base the forecast. Hence, the outcome of the forecast is based upon the knowledge of the experts regarding the problem. On the other hand, quantitative forecasting models make use of the data available to make predictions into the future. The model basically sums up the interesting patterns in the data and presents a statistical association between the past and current values of the variable. Management can use qualitative inputs in conjunction with quantitative forecasts and economic data to forecast sales trends. Qualitative forecasting is useful when there is ambiguous or inadequate data. The qualitative method of forecasting has certain disadvantages such as anchoring events and selective perception. Qualitative forecasts enable a manager to decrease some of this uncertainty to develop plans that are fairly accurate, but still inexact. However, the lack of precision in the development of a qualitative forecast versus a quantitative forecast ensures that no single qualitative technique produces an accurate forecast every time [2,4,7,8,9,10].

In nearly two decades, the fuzzy time series approach has been widely used for its superiorities in dealing with imprecise knowledge (like linguistic) variables in decision making. In the process of forecasting with fuzzy time series models, the fuzzy logical relationship is one of the most critical factors that influence the forecasting accuracy. Many studies seek to deploy neuro-fuzzy inference to the stock market in order to deal with probability. Fuzzy logic is known to be useful for decision-making where there is a great deal of uncertainty as well as vague phenomena, but lacks the learning capability; on the other hand, neural networks are useful in constructing an adaptive system that can learn from historical data, but are not able to process ambiguous rules and probabilistic datasets. It is tedious to develop fuzzy rules and membership functions and fuzzy outputs can be interpreted in a number of ways, making analysis difficult. In addition, it requires a lot of data and expertise to develop a fuzzy system.

Recently, a probabilistic fuzzy set was suggested for forecasting by introducing probability theory into a fuzzy set framework. It changes the secondary MF of type 2 fuzzy into the probability density function (PDF), so it is able to capture the random uncertainties in membership degree. It has the ability to capture uncertainties with fuzzy and random nature. However, the membership functions are difficult to obtain for existing fuzzy approaches of measurement uncertainty. In order to conquer this disadvantage, the cloud model was used to calculate the measurement uncertainty. A cloud is a new, easily visualized concept for uncertainty with well-defined semantics, mediating between the concept of a fuzzy set and that of a probability distribution [11,12,13,14,15,16]. A cloud model is an effective tool in transforming qualitative concepts and their quantitative expressions. The digital characteristics of cloud, expect value (*Ex*), entropy (*En*), and hyper–entropy (He), well integrate the fuzziness and randomness of linguistic concepts in a unified way. Cloud is combined with several cloud drops in which the shape of the cloud reflects the important characters of the quantity concept [17]. The essential difference between the cloud model and the fuzzy probability concept lies in the used method to calculate a random membership degree. Basically, with the three numerical characteristics, the cloud model can randomly generate a degree of membership of an element and implement the uncertain transformation between linguistic concepts and its quantitative instantiations.

Candlestick patterns provide a way to understand which buyer and seller groups currently control the price action. This information is visually represented in the form of different colors on these charts. Recently, several traders and investors have used the traditional Japanese candlestick chart pattern and analyzed the pattern visually for both quantitative and qualitative forecasting [6,7,8,9,10]. Heikin–Ashi candlesticks are an offshoot from Japanese candlesticks. Heikin–Ashi candlesticks use the open–close data from the prior period and the open–high–low–close data from the current period to create a combo candlestick. The resulting candlestick filters out some noise in an effort to better capture the trend.

### 1.1. Problem Statement

The price variation of the stock market is a non–linear dynamic system that deals with non–stationary and volatile data. This is the reason why its modeling is not a simple task. In fact, it is regarded as one of the most challenging modeling problems due to the fact that prices are stochastic. Hence, the best way to predict the stock price is to reduce the level of uncertainty by analyzing the movement of the stock price. The main motivation of our work was the successful prediction of stock future value that can yield enormous capital profits and can avoid potential market risk. Several classical approaches have been evolved based on linear time series models, but the patterns of the stock market are not linear. These approaches lead to inaccurate results, which may be susceptible to highly dynamic factors such as macroeconomic conditions and political events. Moreover, the existing qualitative based methods developed based on fuzzy reasoning techniques cannot describe the data comprehensively, which has greatly limited the objectivity of fuzzy time series in uncertain data forecasting. The most important disadvantage of the fuzzy time series approach is that it needs subjective decisions, especially in the fuzzification stage.

### 1.2. Contribution and Novelty 

The objective of the work presented in this paper is to construct an accurate stock trend prediction model through utilizing a combination of the cloud model, Heikin–Ashi candlesticks, and fuzzy time series (FTS) in a unified model. The purpose of the cloud model is to add the randomness and uncertainty to the fuzziness linguistic definition of Heikin–Ashi candlesticks. FTS is utilized to abstract linguistic values from historical data, instead of numerical ones, to find internal relationship rules. Heikin–Ashi candlesticks were employed to give easier readability of the candle’s features through the reduction of noise, eliminates the gaps between candles, and smoothens the movement of the market.

As far as the authors know, this is the first time that the cloud model has been used in forecasting stock market trends that is unlike the current methods that adopt a fuzzy probability approach for forecasting that requires an expert to define the extra parameters of the probabilistic fuzzy system such as output probability vector in probabilistic fuzzy rules and variance factor. These selected statistical parameters specify the degree of randomness. The cloud model not only focuses on the studies regarding the distribution of samples in the universe, but also try to generalize the point–based membership to a random variable on the interval [0, 1], which can give a brand new method to study the relationship between the randomness of samples and uncertainty of membership degree. More practically speaking, the degree with the aid of three numeric characteristics, by which the transformation between linguistic concepts and numeric values will become possible. 

The outline of the remainder of this paper is as follows. Section 2 presents the background and summary of the state-of-the-art approaches. Section 3 describes the proposed model. The test results and discussion of the meaning are shown in Section 4. The conclusion of this work is given in Section 5.

## 2. Preliminaries and Literature Review

In this section, we summarize material that we need later that includes the cloud model, fuzzy time series, and Heikin–Ashi candlesticks. Finally, some state-of-the-art related works are discussed.

### 2.1. Cloud Model

**Figure 1:**
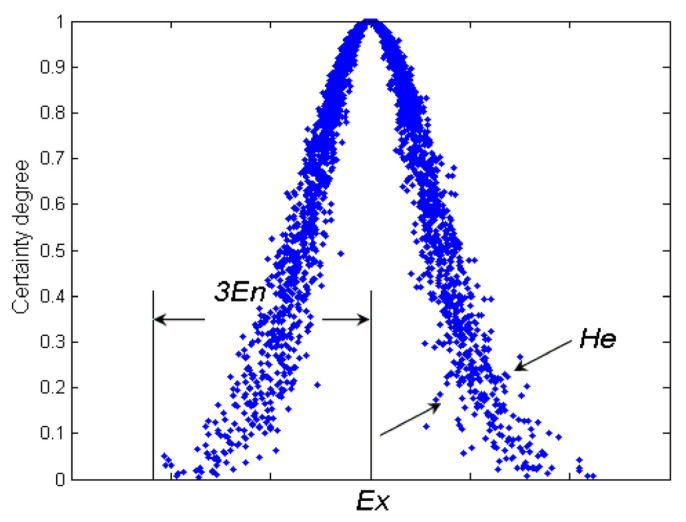
Cloud model.

The cloud model (CM) proposed by Li et al. [17] relies on probability statistics and traditional fuzzy theory [18,19]. The membership cloud model as shown in Figure 1 can mix the fuzziness and randomness to objectively describe the uncertainty of the complex system. This model makes it possible to obtain the range and the distribution of the quantitative data from qualitative information, which is described by linguistic value and effectively transits precise data into appropriate qualitative language value. The digital character of the cloud can be expressed by expected value (*Ex*), entropy (*En*), and hyper entropy (*He*). CM uses *Ex* to represent the qualitative concept and usually is the value of x corresponding to the cloud center. *En* represents the uncertainty measure of the qualitative concept. It measures the ambiguity of the quantitative numerical range. *He* symbols the uncertainty measure of entropy, namely the entropy of entropy, which reflects the dispersion degree of cloud, which appears in the size of the cloud’s thickness [17,18,19,20,21].

**Figure 2:**
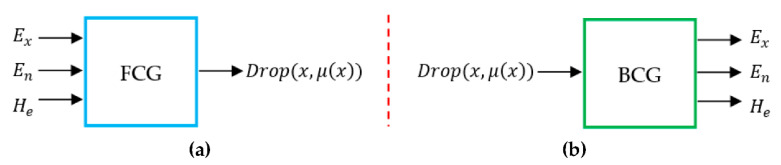
Two different types of cloud generators. (**a**) Forward cloud generator; (**b**) Backward cloud generator.

The theoretical foundation of CM is the probability measure (i.e., the measure function in the sense of probability). On the basis of normal distribution and Gaussian membership function, CMs describe the vagueness of the membership degree of an element by a random variable defined in the universe. Being an uncertain transition way between a qualitative concept described by linguistic terms and its numerical representation, the cloud has depicted such abundant uncertainties in linguistic terms as randomness, fuzziness, and the relationship between them. CM can acquire the range and distributing law of the quantitative data from the qualitative information expressed in linguistic terms. CM has been successfully applied and gives better performance results in several fields such as intelligence control [11], data mining [19], and others. Figure 2 illustrates the types of cloud model (see [11,17] for more details).

### 2.2. The Fuzzy Time Series Model

**Figure 3:**
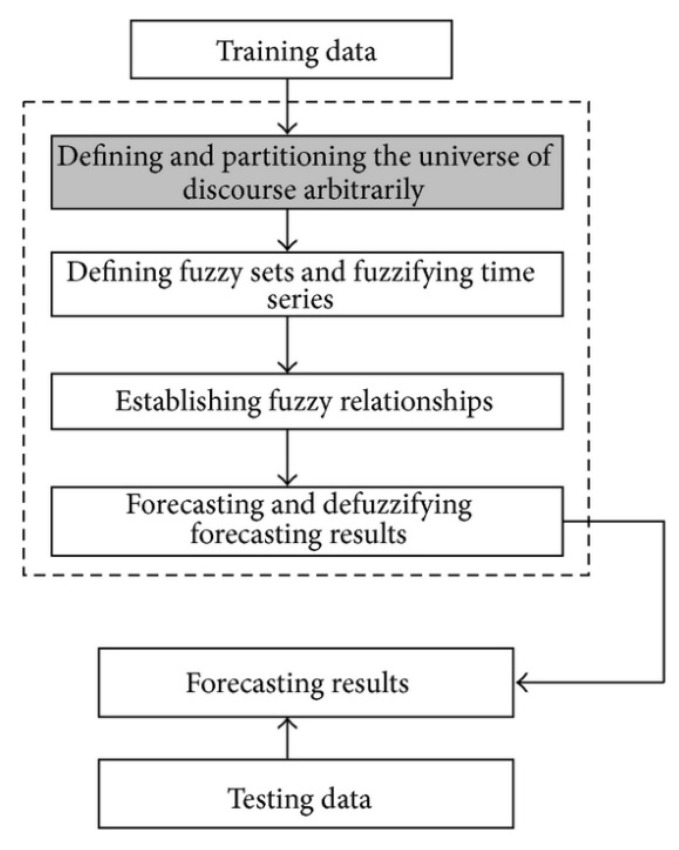
Processes of fuzzy time series forecasting.

Fuzzy time series is another concept to solve forecasting problems in which the historical data are linguistic values. The fuzzy time series has recently received increasing attention because of its capability to deal with vague and incomplete data. There have been a variety of models developed to either improve forecasting accuracy or reduce computation overhead [22]. The fuzzy time series model uses a four–step framework to make forecasts, as shown in Figure 3: (1) define the universe of discourse and partition it into intervals; (2) determine the fuzzy sets on the universe of discourse and fuzzify the time series; (3) build the model of the existing fuzzy logic relationships in the fuzzified time series; and (4) make forecast and defuzzify the forecast values [23,24,25].

Nevertheless, the forecasting performance can be significantly affected by the partition of the universe of discourse. Another issue is the consistency of the forecasting accuracy with the interval length. In general cases, better accuracy can be achieved with a shorter interval length. However, an effective forecasting model should adhere to the consistency principle. In accounting, consistency requires that a company’s financial statements follow the same accounting principles, methods, practices, and procedures from one accounting period to the next. In general, the effect of some parameters in fuzzy time series such as population size, number of intervals, and order of fuzzy time series must be tested and analyzed [26,27].

### 2.3. Heikin–Ashi Candlestick Pattern

**Figure 4:**
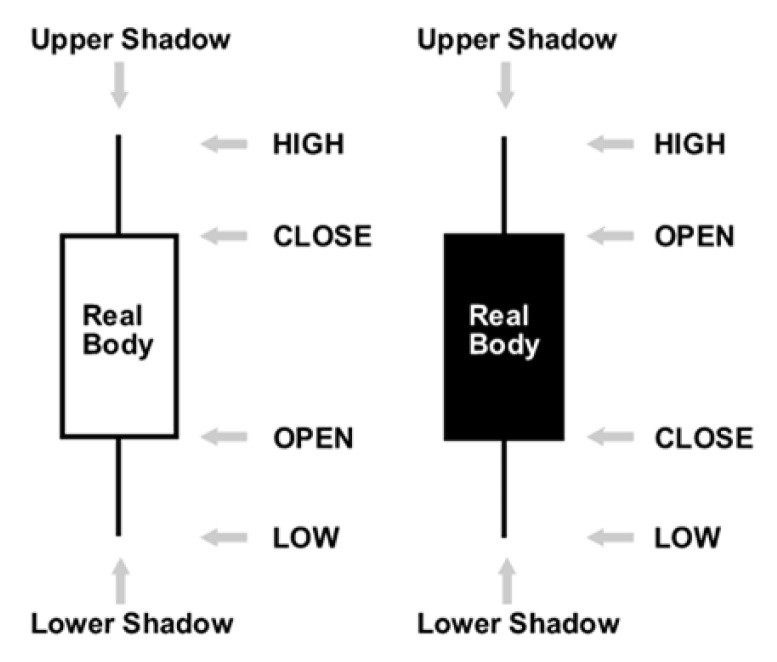
The dark candle and white candle.

The current forecasting models do not contain the qualitative information that would help in predicting the future. Japanese candlesticks are a technical analysis tool that traders use to chart and analyze the price movement of securities. Japanese candlesticks provide more detailed and accurate information about price movements compared to bar charts. They provide a graphical representation of the supply and demand behind each time period’s price action. Each candlestick includes a central portion that shows the distance between the open and the close of the security being traded, the area referred to as the body. The upper shadow is the price distance between the top of the body and the high for the trading period. The lower shadow is the price distance between the bottom of the body and the low for the trading period. The closing price of the security being traded determines whether the candlestick is bullish or bearish. The real body is usually white if the candlestick closes at a higher price than when it opened. In such a case, the closing price is located at the top of the real body and the opening price is located at the bottom. If the security being traded closed at a lower price than it opened for the time period, the body is usually filled up or black in color. The closing price is located at the bottom of the body and the opening price is located at the top. Modern candlesticks now replace the white and black colors of the body with more colors such as red, green, and blue. Traders can choose among the colors when using electronic trading platforms (see Figure 4) [6,7].

Normal candlestick charts are composed of a series of open–high–low–close (OHLC) candles set apart by a time series. The Heikin–Ashi technique shares some characteristics with standard candlestick charts but uses a modified formula of close–open–high–low (COHL). There are a few differences to note between the two types of charts, and are demonstrated by the charts above. Heikin–Ashi has a smoother look as it essentially takes an average of the movement. There is a tendency with Heikin–Ashi for the candles to stay red during a downtrend and green during an uptrend, whereas normal candlesticks alternate colors, even if the price is moving dominantly in one direction. Since Heikin–Ashi takes an average, the current price on the candle may not match the price the market is actually trading at. For this reason, many charting platforms show two prices on the *y*-axis: one for the calculation of the Heikin–Ashi and another for the current price of the asset [7,8,9,10].

### 2.4. Related Work

Researchers that believe in the existence of patterns in a financial time series that make them predictable have centered their work mainly in two different approaches: statistical and artificial intelligence (AI). The statistical techniques most used in financial time series modeling are the autoregressive integrated moving average (ARIMA) and the smooth transition autoregressive (STAR) [2]. On the other hand, artificial intelligence provides sophisticated techniques to model time series and search for behavior patterns: genetic algorithms, fuzzy models, the adaptive neuro-fuzzy inference system (ANFIS), artificial neural networks (ANN), support vector machines (SVM), hidden Markov models, and expert systems, are some examples. Unlike statistical techniques, they are capable of obtaining adequate models for nonlinear and unstructured data. There exists a huge amount of literature that uses AI approaches for time series forecasting [2,4,8]. However, most of them are inaccurate: the computer programs are more effective in syntax analysis than semantic analysis. Furthermore, most of them follow the quantitative forecasting category; qualitative forecasting is useful when there is ambiguous or inadequate data. Most of the current studies were conducted from single time scale features of the stock market index, but it is also meaningful for studying from multiple time scale features [8]. With the development of deep learning, there are many methods based on deep learning used for stock forecasting and have drawn some essential conclusions [3].

In the literature, many studies have used an integrated neuro-fuzzy model to estimate the dynamics of the stock market using technical indicators [3]. This approach integrates the advantages in both the neural and fuzzy models to facilitate reliable intelligent stock value forecasting. However, most of these works did not consider the fractional deviation within a day. Another group of research work utilized hidden Markov models (HMMs) to predict the stock price based on the daily fractional change in the stock share value of intra-day high and low. To benefit from the correlation between the technical indicators and reduce the large dimensionality space, the principal component analysis (PCA) concept was deployed to select the most effective technical indicators among a large number of highly correlated variables. PCA linearly transforms the original large set of input variables into a smaller set of uncorrelated variables to reduce the large dimensionality space.

In addition, some researchers are currently using soft computing techniques (e.g., genetic algorithm) for selecting the most optimal subset of features among a large number of input features, and then selected features are given as input to the machine learning module (e.g., SVM Light software package). Technical analysis is carried out based on technical indicators from the stock to be predicted and also from other stocks that are highly correlated with it. However, the decision is carried out only based on the input feature variables of technical indicators. This leads to prediction errors due to the lack of precise domain knowledge and no consideration of various political and economic factors that affect the stock market other than the technical indicators [3,8].

Song and Chissom [13] suggested a forecasting model using fuzzy time series, which provided a theoretical framework to model a special dynamic process whose observations were linguistic values. The main difference between the traditional time series and fuzzy time series was that the observed values of the former were real numbers while the observed values of the latter were fuzzy sets or linguistic values. Chen et al. [16] presented a new method for forecasting university enrolment using fuzzy time series. Their method is more efficient than the suggested method by Song and Chissom due to the fact that their method used simplified arithmetic operation rather than the complicated MaxMin composition operation. Hwang [22] suggested a new method based on fuzzification to revise Song and Chissom’s method. He used a different triangle fuzzification method to fuzzily crisp values. His method involved determining an interval of extension from both sides of crisp value in triangle membership function to get a variant degree of membership. The results obtained a better average forecasting error. In addition, the influences of factors and variables in a fuzzy time series model such as definition area, number and length of intervals, and the interval of extension in triangle membership function were discussed in detail. More techniques that used fuzzy time series for forecasting can be found in [23,24,25,26,27].

Nison [5] introduced the Japanese candlestick concepts to the Western world. Japanese candlestick patterns are believed to show both quantitative information like price, trend… etc., and qualitative information like the psychology of the market. It considers not only the close values, but also the information on the body of the candlestick can offer an informative summary of the trading sessions [28] and some of its components are predictable [29]. Some researchers have combined technical patterns and candlestick information [30]. In the last decades, several researchers have used Japanese candlesticks in creative forecasting methods [31,32,33,34,35,36]. Lee et al. [31] suggested an expert system with IF–THEN rules to detect candlestick patterns, flag sell, and buy orders with good hit ratios in the Korean market. The authors in [32] displayed Japanese candlestick patterns using fuzzy linguistic variables and knowledge-based by fuzzing both the candle line and the candle lines relationship. In [33], a prediction model was suggested for the financial decision system based on fuzzy candlestick patterns. Lee [34] extended this work through creating and using personal candlestick pattern ontologies to allow different users to have their explanation of a candlestick pattern. Kamo et al. [8,35,36] suggested a model that combined neural networks, committee machines, and fuzzy logic to identify candlestick patterns and generate a market strength weight using fuzzy rules in [35], the type–1 fuzzy logic system in [36], and finally, the type–2 fuzzy logic system in [6].

Naranjo et al. [37] presented a model that used the K-nearest neighbors (KNN) algorithm to forecast the candlestick one day ahead using the fuzzy candlestick representation. Naranjo et al. [38] fuzzified the gap between candles and added it as an extended element in candlesticks patterns. However, Japanese candlestick has contradictory information due to the market’s noise [38]. Recently, the Heikin–Ashi technique modifies the traditional candlestick chart and makes it easier to reduce the noise, eliminate the gaps between candles, and smoothen the movement of the market and let the traders focus on the main trend. The Heikin–Ashi graph is not only more readable than traditional candles, but is also a real trading system [10].

In general, most existing fuzzy time series forecasting models follow fuzzy rules according to the relationships between neighboring states without considering the inconsistency of fluctuations for a related period [38,39,40]. This paper proposes a new perspective to study the problem of prediction, in which inconsistency is quantified and regarded as a key characteristic of prediction rules by utilizing a combination of the cloud model, Heikin–Ashi candlesticks, and fuzzy time series (FTS) in a unified model that can represent both fluctuation trend and fluctuation consistency information.

## 3. Proposed Model

The purpose of the study is to predict and confirm accurate stock future trends due to a lack of insufficient levels of accuracy and certainty. However, there are many problems in previous studies. The main problems in data are uncertainty, noise, non-linearity, non-stationary, and dynamic process of stock prices in time series. In the prediction model, many models are used. The statistical method like the ARMA family is achieved with the trial and error basis iterations. Traders also have problems that include predicting the stock price every day, finding the reversal patterns of the stock price, the difficulty in model parameter tuning, and finally, the gap exists between prediction results and investment decision. Additionally, traditional candlestick patterns have problems such as the definition of the patterns itself being ambiguous and the largest number of patterns.

**Figure 5:**
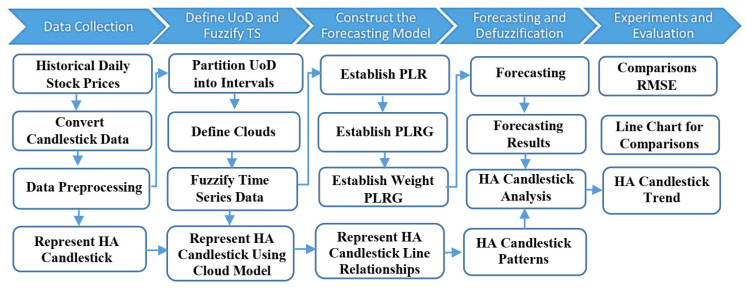
The procedure of the proposed forecasting model.

In order to deal with the above problems, the suggested prediction model uses both cloud model and Heikin–Ashi (HA) candlestick patterns. Figure 5 illustrates the main steps of the suggested model that include preparing historical data, HA candlestick processing, representing the HA candlestick using the cloud model, forecasting the next day price (open, high, low, close) using cloud–based time series prediction, formalizing the next day HA candlestick features, and finally, forecasting the trend and its strong patterns. The following subsection discusses each step in detail [9].

### 3.1. Step 1: Preparing the Historical Data

The publicly available stock market datasets contain historical data on the four price time series for several companies were collected from Yahoo (http://finance.yahoo.com). The dataset specifies the “opening price, lowest price, closing price, highest price, adjusted closing price, and volume” against each date. The data were divided into two parts: the training part and the testing part. The training part from the time series data was used for the formulation of the model while the testing part was used for the validation of the proposed model.

### 3.2. Step 2: Candlestick Data

The first stage in stock market forecasting is the selection of input variables. The two most common types of features that are widely used for predicting the stock market are fundamental indicators and technical indicators. The suggested model used technical indicators that are determined by employing candlestick patterns such as open price, close price, low price, and high price to try to find future stock prices [5,6]. A standard candlestick pattern is composed of one or more candlestick lines. However, the extended candlestick (Heikin–Ashi) patterns have one candlestick line. The HA candlestick uses the modified OHLC values as candlesticks that are calculated using [5]:

(1) $$\begin{array}{c} Ha_{Close}=\frac{\left(Open\;+\;High\;+\;Low\;+\;Close\right)}{4}\\ Ha_{Open}=\frac{(Ha_{Open}\left(Previous\;Bar\right)\;+\;Ha_{Close}\left(Previous\;Bar\right)}{2}\\ \;Ha_{High}=Max\left(High,\;Ha_{Open},\;Ha_{Close}\right)\\ Ha_{Low}=Min\left(Low,\;Ha_{Open},\;Ha_{Close}\right) \end{array}\}$$

Herein, each candlestick line has the following parameters: length of the upper shadow, length of the lower shadow, length of the body, color, open style, and close style. The open style and close style are formed by the relationship between a candlestick line and its previous candlestick line. The crisp value of the length of the upper shadow, length of the lower shadow, length of the body, and color play an important role in identifying a candlestick pattern and determining the efficiency of the candlestick pattern. The candlestick parameters are directly calculated using [9,10].

(2) $$\begin{array}{c} HaL_{Body}=\frac{Max\left(Ha_{open}\;,\;Ha_{Close}\right)\;-\;Min\left(Ha_{open},\;Ha_{Close}\right)}{Ha_{open}}\times 100\\ HaL_{UpperShadow}=\frac{Ha_{High}\;-\;Max\left(Ha_{open},\;Ha_{Close}\right)}{Open}\times 100\\ \;HaL_{LowerShadow}=\frac{Min\left(Ha_{open},\;Ha_{Close}\right)\;-\;Ha_{Low}}{Ha_{open}}\times 100\\ Ha_{Color}=Ha_{Close}\;-\;Ha_{open} \end{array}\}$$

where *HaL* indicates the length of the body, upper shadow, or lower shadow of the HA candlestick. The *Ha_COLOR_* parameter represents the mean body color of the HA candlestick. Heikin–Ashi candlesticks are similar to conventional ones, but rather than using opens, closes, highs, and lows, they use average values for these four price metrics.

In stock market prediction, the quality of data is the main factor because the accuracy and the reliability of the prediction model depends upon the quality of data. Any unwanted anomalies in the dataset are known as noise. Outliers are the set of observations that do not obey the general behavior of the dataset. The presence of noise and outliers may result in poor prediction accuracy of forecasting models. The data must be prepared so that it covers the range of inputs for which the network is going to be used. Data pre-processing techniques attempt to reduce errors and remove outliers, hence improving the accuracy of prediction models. The purpose of HA charts is to filter noise and provide a clearer visual representation of the trend. Heikin–Ashi has a smoother look, as it is essentially taking an average of the movement [9,10].

### 3.3. Step 3: Cloud Model-Based Candlestick Representation

**Figure 6:**
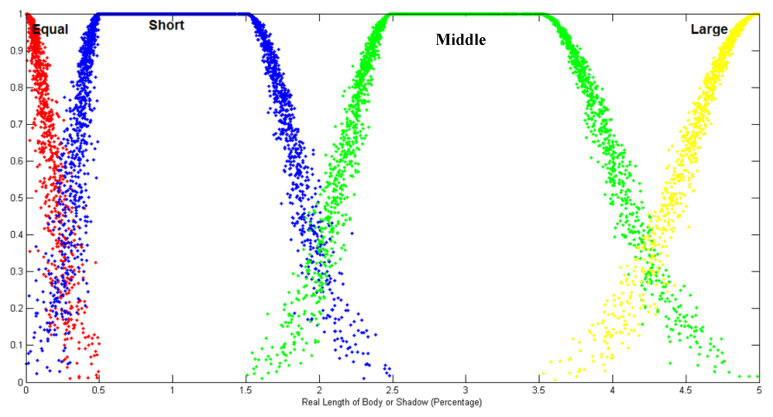
The membership function of the body and shadow length based on the cloud model.

There is no crisp value to define the length of body and shadow in the HA candlestick; these variables are usually described as imprecise and vague. Herrin, to transform crisp candlestick parameters (HA quantitative values) to linguistic variables to define the candlestick (qualitative value), the cloud model was used. To achieve this goal, fuzzy HA candlestick pattern ontology was built that contains [4,8]:

- Candlestick Lines: Four fuzzy linguistic variables, equal, short, middle, and long, were defined to indicate the cloud model of the shadows and the body length. Figure 6 shows the membership function of the linguistic variables based on the cloud model, then used the maximum μ(x) to determine its linguistic variable. The ranges of body and shadow length were set to (0, p) to represent the percentage of the fluctuation of stock price. The parameter value of each fuzzy linguistic variable was set as stated in [8]. See [8] for more details regarding the rationale of using these values. These fuzzy linguistic variables are defined as:

(3) $$Equal\left(x:a,b\right)=\{\begin{array}{cc} 0 & x<a\\ exp\left(-\frac{1}{2}{\left(\frac{x-Ex}{En}\right)}^{2}\right) & a\leq x\leq b\\ 0 & x>b \end{array}$$

(4) $$\mathrm{Short}/\mathrm{Middle}\left(x:a,b,c,d\right)=\{\begin{array}{cc} 0 & x<a\\ exp\left(-\frac{1}{2}{\left(\frac{x-{Ex}_{1}}{{En}_{1}}\right)}^{2}\right) & a\leq x\leq b\\ 1 & b<x<c\\ exp\left(-\frac{1}{2}{\left(\frac{x-{Ex}_{2}}{{En}_{2}}\right)}^{2}\right) & c\leq x\leq d\\ 0 & x>d \end{array}$$

(5) $$Large\left(x:a,b\right)=\{\begin{array}{cc} 0 & x<a\\ exp\left(-\frac{1}{2}{\left(\frac{x-Ex}{En}\right)}^{2}\right) & a\leq x\leq b\\ 1 & x>b \end{array}$$

The body color $Body_{Color}$ is also an import feature of a candlestick line. It is defined by three terms Black, White, and Doji. A Doji term is defined to describe the situation where the open price equals the close price. In this case, the height of the body is 0, and the shape is represented by a horizontal bar. The definition of body color is defined as [10]:

(6) $$\begin{array}{c} If\left(Open-Close\right)>0\;Then\;Body_{Color}=Black\\ If\left(Open-Close\right)<0\;Then\;Body_{Color}=White\\ If\left(Open-Close\right)=0\;Then\;Body_{Color}=Doji \end{array}\}$$

**Figure 7:**
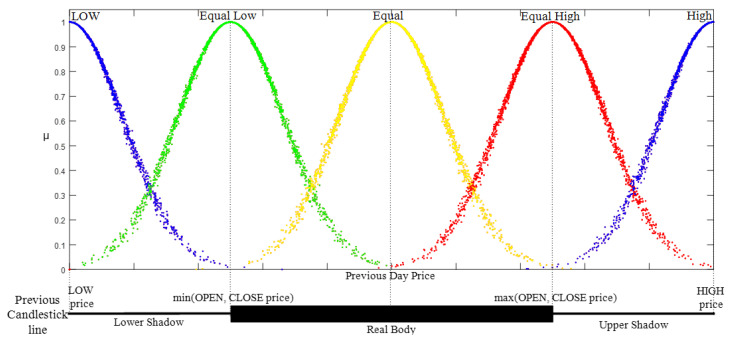
The membership function of the open and close styles based on the cloud model.

- Candlestick Lines Relationships: This defines the place of the HA candlestick with the previous one to form open style and close style linguistic variables. In general, merging the description of the candlestick line and HA candlestick line relationship can create a HA candlestick pattern that is completely defined. Herein, five linguistic variables were defined to represent the relationship style (X style): low, equal low, equal, equal high, and high. Their membership function follows half bell cloud defined in Equation (7). Additionally, the parameter value of each fuzzy linguistic variable was set as stated in [8]. Figure 7 shows the membership function of the linguistic variable based on the cloud model:

(7) $$X\_Style\left(x:a,b\right)=\{\begin{array}{cc} 0 & x<a\\ exp\left(-\frac{1}{2}{\left(\frac{x-Ex}{En}\right)}^{2}\right) & a\leq x\leq \\ 0 & x>b \end{array}b$$

In our case, membership cloud function (forward normal cloud generator) converts the statistic results to fuzzy numbers, and constructs the one–to–many mapping model. The input of the forward normal cloud generator is three numerical characteristics of a linguistic term, (*Ex*, *En*, *He*), and the number of cloud drops to be generated, *N*, while the output is the quantitative positions of *N* cloud drops in the data space and the certain degree that each cloud drop can represent the linguistic term. The algorithm in detail is:

- Produce a normally distributed random number *En*’ with mean *En* and standard deviation *He*;
- Produce a normally distributed random number x with mean *Ex* and standard deviation *En*’;
- Calculate Y = $exp\left(-\frac{1}{2}{\left(\frac{x-Ex}{En}\right)}^{2}\right)$
- Drop (x,y) is a cloud drop in the universe of discourse; and
- Repeat step 1–4 until N cloud drops are generated.

Expectation value (*Ex*) at the center-of-gravity positions of cloud drops is the central value of distribution. Entropy (*En*) is the fuzzy measure of qualitative concept that describes the uncertainty and the randomness. The larger the entropy, the larger the acceptable interval of this qualitative concept, which represents that this conception is more fuzzy. Hyper entropy (He) is the uncertain measure of qualitative concept that describes the dispersion. The larger the hyper entropy, the thicker the shape of the cloud, which shows that this conception is more discrete [20,21].

– Forecast the next day price (open, high, low, close)

**Figure 8:**
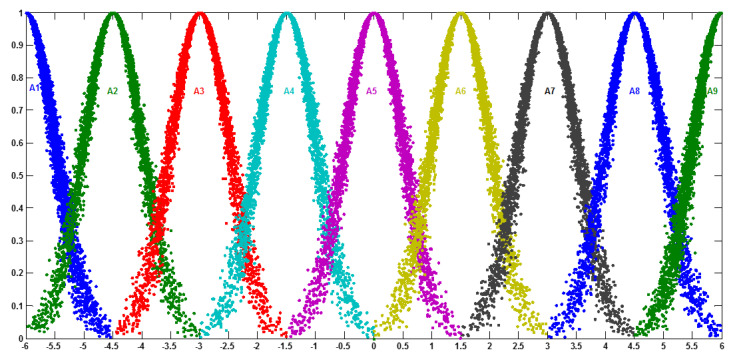
The clouds of the linguistic terms.

In the fuzzy candlestick pattern approach, the measured values are the open, close, high, and low price of trading targets in a specific time period. The features of the trading target price fluctuation are represented by the fuzzy candlestick pattern. The classification rules of fuzzy candlestick patterns can be determined by the investors or the computer system. In general, using a candlestick pattern approach for financial time series prediction consists of the following steps [21]:

- Partitioning the universe of discourse into intervals: In this case, after preparing the historical data and defining the range of the universe of discourse (UoD), open, high, low, and close prices should be established as a data price set for each one. Then, for each data price set, the variation percentage between two prices on time t and time t+n is calculated $(\left(Close_{t+n}-Close_{t}\right)/Close_{t})\times 100\;$to partition the universe of discourse dataset into intervals. Based on the variation, the minimum variation $D_{min}$ and the maximum variation $D_{max}\;$are determined that define $U=\left[D_{min}-D_{1},\;D_{max}+D_{2}\right]$, where $D_{1}$ and $D_{2}\;$are suitable positive numbers.
- Classifying the historical data to its cloud: The next step determines the linguistic variables represented by clouds (see Figure 8) to describe the degree of variation between data of time t and time t+n and defined it as a set of linguistic terms. Table 1 shows the digital characteristics of the cloud member function (*Ex*, *En*, *He*) for each linguistic term.
- Building the predictive logical relationships (PLR): The model builds the PLR to carry on the soft inference $\;A_{t-1}\to A_{t}$, where $A_{t-1}$ and $A_{t}$ are clouds representing linguistic concepts, by searching all clouds in time series with the pattern ($A_{t-1}\to A_{t}$).
- Building of predictive linguistic relationship groups (PLRG): In the training dataset, all PLRs with the same “current state” will be grouped into the same PLRG. If $A_{1}$, $A_{2}$,⋯, $A_{m}$ is the “current state” of one PLR in the training dataset and there are *r* PLRs in the training dataset as$\;A_{1}\to A_{1}$; $\;A_{1}\to A_{2}$; …. ;$\;A_{1}\to A_{m}$, the *r* PLRs can be grouped into the same PLRG, as $A_{1}\to A_{1},A_{2},\;\ldots .,\;A_{m}$. Then, assign the weight elements for each PLRG. Assume $\;A_{i}\;$has $\;n_{1}$ relationships with $\;A_{1}$, $\;n_{2}$ relationships with $\;A_{2}$, and so on. The weight values (*w*) can be assigned as *w*_i_ = (number of recurrence of *A*_i_)/(total number of PLRs).

- Calculating the predicted value via defuzzification: Then the model forecasts the next day (open, high, low, close) prices through defuzzification and calculates the predicted value at time *t* P(*t*) by following the rule:
  ✔ Rule 1: If there is only one PLR in the PLRG, ($\;A_{1}\to A_{i}$) then,
    (8) $$P\left(t\right)=\frac{Ex_{i}+S\left(t-1\right)}{2}$$
  ✔ Rule 2: If there is *r* PLR in the PLRG, ($\;A_{1}\to A_{1},A_{2},\;\ldots .,\;A_{p}$) then,
    (9) $$P\left(t\right)=\frac{1}{2}\left(\left(\frac{\left(n_{1}\times Ex_{1})+(n_{2}\times Ex_{2})+\ldots +(n_{p}\times Ex_{p}\right)}{n_{1}+n_{1}+\ldots +n_{p}}\right)+S\left(t-1\right)\right)$$
  ✔ Rule 3: If there is no PLR in the PLRG, ($\;A_{1}\to \#$) where the symbol “#” denotes an unknown value; then apply Equation (8). $\;Ex_{i\;}$is the expectation of the Gaussian cloud$\;C_{i}$ corresponding to$\;A_{i}$,$\;n_{i}$ is the number of$\;A_{i}$ appearing in the PLRG, 1 ≤ *i* ≤ *r*, and S(*t* − 1) denotes the observed value at time *t* – 1.

- Transforming the forecasting results (open, high, low, and close) to the next HA candlestick. through the following rules [9]:
  ✔ Rule 1: **If** $Body_{Color}$ is White and $HaL_{Body}$ is Long **Then**, UP Trend.
  ✔ Rule 2: **If** $Body_{Color}$ is Black and $HaL_{Body}$ is Long **Then**, Down Trend.
  ✔ Rule 3: **If** $Body_{Color}$ is White and $HaL_{Body}$ is Long and $HaL_{LowerShadow}$ is Equal **Then**, Strong UP Trend.
  ✔ Rule 4: **If** $Body_{Color}$ is Black and $HaL_{Body}$ is Long and $HaL_{UpperShadow}$ is Equal **Then**, Strong Down Trend.
  ✔ Rule 5: **If** ($HaL_{Body}$ is Equal) and ($HaL_{UpperShadow}$ & $HaL_{LowerShadow}$) is Long **Then** Change of Trend.
  ✔ Rule 6: **If** ($HaL_{Body}$ is Short) and ($HaL_{UpperShadow}$ & $HaL_{LowerShadow}$) is not Equal **Then**, Consolidation Trend.
  ✔ Rule 7: **If** ($HaL_{Body}$ is Short or Equal) and ($Ha_{Open}$_Style and $Ha_{Close}$_Style) is (Low_Style or EqualLow_Style) and $HaL_{UpperShadow}$ is Equal **Then** Weaker Trend.

## 4. Experimental Results

In order to test the efficiency and validity of the proposed model, the model was implemented in MATLAB language. The prototype verification technique was built in a modular fashion and has been implemented and tested in a Dell™ Inspiron™ N5110 Laptop machine, Dell computer Corporation, Texas, which had the following features: Intel(R) Core(TM) i5–2410M CPU@ 2.30GHz, and 4.00 GB of RAM, 64–bit Windows 7. A dataset composed of real-time stocks series of the NYSE (New York Stock Exchange) was used in the experimentation. The dataset had 13 time series of NYSE companies, each one with the four prices (open, high, low, and close). Time series were downloaded from the Yahoo finance website (http://finance.yahoo.com), Table 2 shows the companies’ names, symbol, and starting date and ending date for the selected dataset. The dataset was divided into 2/3 for training and the other 1/3 for testing.

In the proposed forecasting model, the parameters were set as follows: the ranges of body (p) and shadow length were set to (0, 14) to represent the percentage of the fluctuation of stock price because the varying percentages of the stock prices are limited to 14 percent in the Taiwanese stock market, for example. It should be noted that although we limited the fluctuation of body and shadow length to 14 percent, in other applications, the designer can change the range of the fluctuation length to any number [4]. The four parameters (a–d) of the function to describe the linguistic variables SHORT and MIDDLE were (0, 0.5, 1.5, 2.5) and (1.5, 2.5, 3.5, 5). The parameters (a, b) that were used to model the EQUAL fuzzy set were equal to (0, 0.5). Regarding the two parameters $D_{1}$ and $D_{2}$, which are used to determine the UOD, we can set $D_{1}$ = 0:17 and $D_{2}$ = 0:34, so the UoD can be represented as [6,8]. Finally, the number of drops in the cloud model used to build the membership function is usually equal to the number of samples in the dataset to describe the data efficiently. The mean squared error (MSE) and mean absolute percentage error (MAPE) that are used by academicians and practitioners [4,21] were used to evaluate the accuracy of the proposed method. Table 3, Table 4, Table 5 and Table 6 show the output of applying each model step for the Yahoo dataset.

(10) $$MSE=\frac{\sum_{i=1}^{n}{\left(Forcasted\;Value-Actual\;Value\right)}^{2}}{n}$$

(11) $$MAPE=\frac{1}{n}\overset{n}{\underset{i=1}{\sum }}\left|\frac{{\left(Actual\;Value\right)}_{i}-{\left(Forcasted\;Value\right)}_{i}}{{\left(Actual\;Value\right)}_{i}}\right|$$

The suggested model was verified with respect to the RMS on both the training and testing data. The predicted prices of the model were found to be correct and close to the actual prices. There was a clear difference between the MSE values for the training and testing data, showing that the model was overfitting the training data as the error on the training dataset was minimized. The reason for this is that the model was not as generalized and was specialized to the structure in the training dataset. Using cross validation represents one possible way to handle overfitting, and using multiple runs of cross validation is better again. The model RMS is summarized in Table 7.

Table 8 shows the comparison results between our two versions of the suggested model: the first one uses open, high, low, and close price as the initial price in the cloud FTS model (Cloud FTS) and the second method uses HaOpen, HaHigh, HaLow, and HaClose prices as the initial price in the cloud FTS model (HA Cloud FTS), and other two standard Song fuzzy time series (FTS) [13,14] and Yu weighted fuzzy time series (WFTS) models [23]. In Song’s studies, the fuzzy relationships were treated as if they were equally important, which might not have properly reflected the importance of each individual fuzzy relationship in forecasting. In Yu’s study, it is recommended that different weights be assigned to various fuzzy relationships. From Table 8, the MSE of the forecasting results of the proposed model was smaller than that of the other methods for all datasets. That is, the proposed model could obtain a higher forecasting accuracy rate for forecasting stock prices than the Song FTS and Yu WFTS models. In general, the MSE values changed according to the nature of each dataset. It can be noted from the table that the Wells Fargo dataset yielded the best results in terms of RMS for both the training and testing data. In general, the Wells Fargo dataset is a small dataset (2,313 row and 12 column) that is probably linearly separable, so it produced high accuracy. This is a bit difficult to accomplish with larger data, so the algorithm produced lower accuracy.

One possible explanation of these results is that, compared with standard models that use FTS only, utilizing FTS with the cloud model helps to automatically produces random membership grades of a concept through a cloud generator. In this way, the membership functions are built based on the characteristics of the data instead of traditional fuzzy–based forecasting methods that depend on the expert. From the point of view of the importance of using HA candlesticks with the cloud model for forecasting, utilizing the HA candlesticks showed significant features that could identify market turning points and also the direction of the trend that helps improve prediction accuracy.

**Figure 9:**
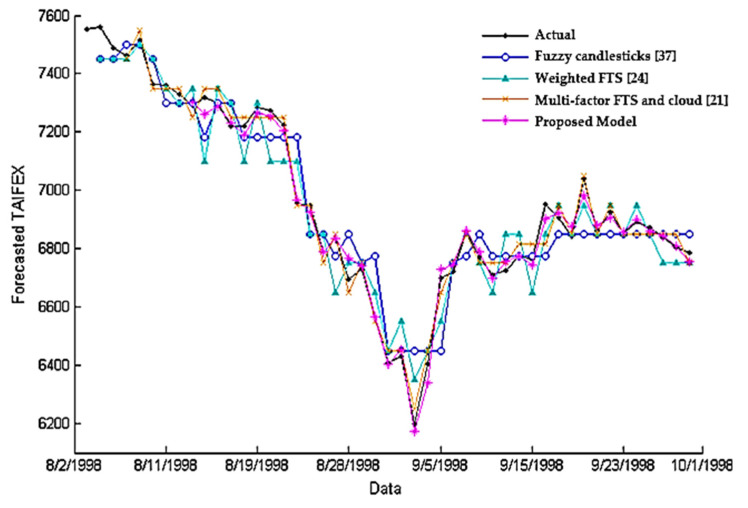
Comparison of the forecasting values of different methods.

The last set of experiments was fulfilled to validate the efficiency of the suggested model compared to state-of-the-art models listed in Figure 9 using the Taiwan Capitalization Weighted Stock Index (TAIEX). The data used for comparison were obtained from a website https://www.twse.com.tw/ that provided the stock prices prevailing at the NASDAQ stock quotes. As shown in Figure 9, the proposed model can perform effective prediction where the predicted stock price closely resembles the actual price in the stock market. The MSE of the suggested model was 665.40 compared with 1254.90, 4530.45, and 4698.78 for the other methods, respectively. Clearly, the suggested model had a smaller MSE than the previous methods. One of the reasons for this result is due to the merging between the cloud model and HA candlesticks, which makes it possible to account for the vagueness and uncertainty of the pattern features based on data characteristics.

## 5. Conclusions

In recent years, mathematical and computational models from artificial intelligence have been used for forecasting. Knowing about future values and the stock market trend has attracted a lot of attention by researchers, investors, financial experts, and brokers. This work analyzed stock trading due to its high non-linear, uncertain, and dynamic data over time. Therefore, this paper presented a Japanese candlestick-based cloud model for stock price prediction that minimizes the investor risk while investing money in the stock market. The proposed work presented an enhanced fuzzy time series forecasting model based on the cloud model and Heikin–Ashi Japanese candlestick to predict and confirm the accurate stock trends. The objective of this model was to handle qualitative forecasting and not quantitative only. The experimental result showed that using HA Cloud FTS and Cloud FTS had a lower average than the other methods used in the literature. This low average proves the high accuracy of the proposed model. HA Cloud FTS provided a MSE = 0.779 for the training data and 0.176 for the test data and Cloud FTS gave a MSE of 0.939 for the training data and 0.240 for the test data; these results mean that the HA Cloud FTS method, which uses HaOpen, HaHigh, HaLow, HaClose prices as the initial price, has a significant improvement in stock market trend prediction. Future work includes embedding Neutrosophic logic to enhance qualitative forecasting.

## Figures and Tables

**Table 1:** The digital characteristics of cloud member function for each linguistic term.

Price Variation		[−6, −4.5]	[−6, −3]	[−4.5, −1.5]	[−3, 0]	[−1.5, 1.5]	[0, 3]	[1.5, 4.5]	[3, 6]	[4.5, 6]
Linguistic Terms		A1 Extreme Decrease	A2 Large Decrease	A3 Normal Decrease	A4 Small Decrease	A5 No Change	A6 Small Increase	A7 Normal Increase	A8 Large Increase	A9 Extreme Increase
CG	*Ex*	−6	−4.5	−3	−1.5	0	1.5	3	4.5	6
	*En*	0.5	0.5	0.5	0.5	0.5	0.5	0.5	0.5	0.5
	*He*	0.05	0.05	0.05	0.05	0.05	0.05	0.05	0.05	0.05

**Table 2:** Selected time series datasets.

Company	Symbol	from	to
Boeing Company	BA	02/01/1962	27/06/2018
Bank of America	BAC	03/01/2000	12/12/2014
DuPont	DD	03/01/2000	12/12/2014
Ford Motor Co.	F	03/01/2000	12/12/2014
General Electric	GE	03/01/2000	12/12/2014
Hewlett–Packard	HPQ	03/01/2000	12/12/2014
Microsoft	MSFT	03/01/2000	12/12/2014
Monsanto	MON	18/10/2000	12/12/2014
Toyota Motor	TM	03/01/2000	12/12/2014
Wells Fargo	WFC	01/06/1972	27/06/2018
Yahoo	YHOO	03/01/2005	12/12/2014
Exxon Mobil	XOM	02/01/1970	21/05/2018
Walt Disney	DIS	02/01/1962	27/06/2018

**Table 3:** Heiken–Ashi candlestick patterns derived from Yahoo training data.

Date	Open	High	Low	Close	HA Open	HA High	HA Low	HA Close	HA Body	HA Upper Shadow	HA Lower Shadow	HA Color	HA Open Style	HA Close Style
10/01/2005	36.00	36.76	35.51	36.32	36.16	36.76	35.51	36.15	EQUAL	SHORT	SHORT	BLACK	HIGH	HIGH
11/01/2005	36.31	36.58	35.39	35.66	36.15	36.58	35.39	35.99	SHORT	SHORT	SHORT	BLACK	EQUAL_HIGH	EQUAL_HIGH
12/01/2005	35.88	36.18	34.80	36.14	36.07	36.18	34.80	35.75	SHORT	SHORT	MIDDLE	BLACK	EQUAL_HIGH	EQUAL_HIGH
13/01/2005	36.12	36.32	35.26	35.33	35.91	36.32	35.26	35.76	SHORT	SHORT	SHORT	BLACK	EQUAL_HIGH	EQUAL_HIGH
14/01/2005	35.86	36.70	35.83	36.70	35.83	36.70	35.83	36.27	SHORT	SHORT	EQUAL	WHITE	EQUAL_HIGH	HIGH
…	…	…	…	…	…	…	…	…	…	…	…	…	…	…
…	…	…	…	…	…	…	…	…	…	…	…	…	…	…
28/11/2014	51.87	52.00	51.64	51.74	51.73	52.00	51.64	51.81	EQUAL	SHORT	EQUAL	WHITE	EQUAL_HIGH	EQUAL_HIGH
01/12/2014	51.43	51.43	49.66	50.10	51.77	51.77	49.66	50.66	MIDDLE	EQUAL	SHORT	BLACK	EQUAL_HIGH	LOW
02/12/2014	50.27	51.12	50.01	50.67	51.21	51.21	50.01	50.52	SHORT	EQUAL	SHORT	BLACK	EQUAL_HIGH	EQUAL_HIGH
03/12/2014	50.71	50.97	50.20	50.28	50.87	50.97	50.20	50.54	SHORT	EQUAL	SHORT	BLACK	EQUAL_HIGH	EQUAL_HIGH
04/12/2014	50.19	50.67	49.90	50.41	50.70	50.70	49.90	50.29	SHORT	EQUAL	SHORT	BLACK	EQUAL_HIGH	EQUAL_HIGH
05/12/2014	51.03	51.25	50.51	50.99	50.50	51.25	50.50	50.95	SHORT	SHORT	EQUAL	WHITE	EQUAL_HIGH	HIGH
08/12/2014	50.52	50.90	49.22	49.62	50.72	50.90	49.22	50.07	SHORT	SHORT	SHORT	BLACK	LOW	LOW
09/12/2014	48.75	50.53	48.29	50.51	50.39	50.53	48.29	49.52	SHORT	SHORT	MIDDLE	BLACK	EQUAL_HIGH	EQUAL_HIGH
10/12/2014	50.33	50.69	49.19	49.21	49.96	50.69	49.19	49.86	EQUAL	SHORT	SHORT	BLACK	EQUAL_HIGH	EQUAL_HIGH

**Table 4:** Yahoo dataset, one day variations, and its cloud.

Date	Open	High	Low	Close	One Day Variations	Cloud	One Day Variations	Cloud	One Day Variations	Cloud	One Day Variations	Cloud
					Open		High		Low		Close	
	O	H	L	C	O		H		L		C	
03/01/2005	38.36	38.9	37.65	38.18	0.00		0.00		0.00		0.00	
04/01/2005	38.45	38.54	36.46	36.58	0.23	A5	−0.93	A4	−3.16	A3	−4.19	A2
05/01/2005	36.69	36.98	36.06	36.13	−4.58	A2	−4.05	A2	−1.10	A4	−1.23	A4
06/01/2005	36.32	36.5	35.21	35.43	−1.01	A4	−1.30	A4	−2.36	A4	−1.94	A4
07/01/2005	35.99	36.46	35.41	35.96	−0.91	A4	−0.11	A4	0.57	A5	1.50	A6
10/01/2005	36.00	36.76	35.51	36.32	0.03	A4	0.82	A6	0.28	A5	1.00	A6
….	…	…	…..	…	…..	….	…..	….	…..	….	…..	….
….	…	…	…..	…	…..	….	…..	….	…..	….	…..	….
10/12/2014	50.33	50.69	49.19	49.21	3.24	A7	0.32	A5	1.86	A6	−2.57	A4
11/12/2014	49.54	50.58	49.43	49.94	−1.57	A4	−0.22	A4	0.49	A5	1.48	A6

**Table 5:** The PLR results.

Date	Open PLR	High PLR	Low PLR	Close PLR
03/01/2005				
04/01/2005	A_5_ → A2	A4 → A2	A3 → A4	A2 → A4
05/01/2005	A2 → A4	A2 → A4	A4 → A4	A4 → A4
06/01/2005	A4 → A4	A4 → A4	A4 → A5	A4 → A6
07/01/2005	A4 → A4	A4 → A6	A5 → A5	A6 → A6
….	….	….	….	….
….	….	….	….	….
09/12/2014	A2 → A7	A4 → A5	A4 → A6	A6 → A4
10/12/2014	A7 → A4	A5 → A4	A6 → A5	A4 → A6
11/12/2014	A4 → A4	A4 → A6	A5 → A4	A6 → A5

**Table 6:** PLRG for close PLR.

Close		To									Total Count
		A1	A2	A3	A4	A5	A6	A7	A8	A9	
From	A1	0	2	0	14	1	2	2	0	3	24
	A2	2	7	3	21	4	16	6	6	5	70
	A3	0	1	1	15	3	7	2	2	1	32
	A4	6	30	15	370	79	170	67	29	17	783
	A5	4	3	3	100	22	28	11	7	1	179
	A6	5	10	3	152	42	64	32	13	6	327
	A7	3	9	3	68	19	21	4	6	3	136
	A8	0	3	2	25	8	15	10	7	3	73
	A9	4	5	2	18	1	4	2	3	5	44
											1668

**Table 7:** Average MSE of the suggested model for all dataset.

MSE	Open	High	Low	Close
Training Data	0.09	0.19	0.16	0.20
Testing Data	0.03	0.07	0.07	0.07

**Table 8:** MSE Comparison for CLOSE price prediction between HA Cloud FTS, Cloud FTS, Yu WFTS and Song.

	MSE	HA Cloud FTS		Cloud FTS		Yu WFTS [23]		Song FTS [14]	
Company		Train	Test	Train	Test	Train	Test	Train	Test
Boeing Company	BA	0.048	0.672	0.078	0.960	5.290	3.460	5.954	3.725
Bank of America	BAC	0.941	0.023	1.124	0.029	6.503	2.592	2.756	0.960
DuPont	DD	0.270	0.116	0.397	0.152	5.336	2.496	14.516	7.076
Ford Motor Co.	F	0.168	0.020	0.203	0.026	5.905	2.690	4.080	1.588
General Electric	GE	3.204	0.023	3.423	0.036	8.526	2.403	9.425	2.074
Hewlett–Packard	HPQ	1.392	0.096	1.769	0.130	7.182	2.756	6.605	2.372
Microsoft	MSFT	0.740	0.048	0.922	0.068	5.905	2.403	7.129	2.372
Monsanto	MON	1.904	0.314	2.528	0.476	8.009	3.028	6.052	1.588
Toyota Motor	TM	1.166	0.449	1.369	0.504	6.300	2.856	19.272	9.303
Wells Fargo	WFC	0.023	0.102	0.040	0.144	4.928	2.624	3.133	1.638
Yahoo	YHOO	0.203	0.073	0.250	0.090	5.664	2.624	6.052	2.496
Exxon Mobil	XOM	0.040	0.221	0.068	0.314	4.580	2.560	6.656	3.572
Walt Disney	DIS	0.023	0.130	0.036	0.194	5.198	2.723	4.580	2.250
AVERAGE		0.779	0.176	0.939	0.240	6.102	2.709	7.400	3.155
